# Prevalence of Influenza B/Yamagata Viruses From Season 2012/2013 to 2021/2022 in Italy as an Indication of a Potential Lineage Extinction

**DOI:** 10.1111/irv.13359

**Published:** 2024-09-10

**Authors:** Serena Marchi, Marco Bruttini, Giovanna Milano, Ilaria Manini, Maria Chironna, Elena Pariani, Alessandro Manenti, Otfried Kistner, Emanuele Montomoli, Nigel Temperton, Claudia Maria Trombetta

**Affiliations:** ^1^ Department of Molecular and Developmental Medicine University of Siena Siena Italy; ^2^ Tuscan Centre of Precision Medicine, Department of Medicine, Surgery and Neurosciences University of Siena Siena Italy; ^3^ Department of Interdisciplinary Medicine University of Bari Aldo Moro Bari Italy; ^4^ Department of Biomedical Sciences for Health University of Milan Milan Italy; ^5^ VisMederi srl Siena Italy; ^6^ VisMederi Research srl Siena Italy; ^7^ Viral Pseudotype Unit, Medway School of Pharmacy University of Kent and Greenwich Chatham Maritime Kent UK

**Keywords:** humoral immunity, influenza B virus, Yamagata lineage

## Abstract

**Background:**

Influenza B/Yamagata viruses exhibited weak antigenic selection in recent years, reducing their prevalence over time and requiring no update of the vaccine component since 2015. To date, no B/Yamagata viruses have been isolated or sequenced since March 2020.

**Methods:**

The antibody prevalence against the current B/Yamagata vaccine strain in Italy was investigated: For each influenza season from 2012/2013 to 2021/2022, 100 human serum samples were tested by haemagglutination inhibition (HAI) assay against the vaccine strain B/Phuket/3073/2013. In addition, the sequences of 156 B/Yamagata strains isolated during the influenza surveillance activities were selected for analysis of the haemagglutinin genome segment.

**Results:**

About 61.9% of the human samples showed HAI antibodies, and 21.7% had protective antibody levels. The prevalence of antibodies at protective levels in the seasons between the isolation of the strain and its inclusion in the vaccine was between 11% and 25%, with no significant changes observed in subsequent years. A significant increase was observed in the 2020/2021 season, in line with the increase in influenza vaccine uptake during the pandemic. Sequence analysis showed that from 2014/2015 season onward, all B/Yamagata strains circulating in Italy were closely related to the B/Phuket/2013 vaccine strain, showing only limited amino acid variation.

**Conclusions:**

A consistent prevalence of antibodies to the current B/Yamagata vaccine strain in the general population was observed. The prolonged use of a well‐matched influenza vaccine and a low antigenic diversity of B/Yamagata viruses may have facilitated a strong reduction in B/Yamagata circulation, potentially contributing to the disappearance of this lineage.

## 1 Introduction

Infection with influenza B virus (IBV) accounts for around a quarter of the annual influenza burden. Since 1985, two genetically and antigenically distinct lineages of IBVs, referred to as B/Victoria/2/1987 and B/Yamagata/16/1988 (hereafter B/Victoria and B/Yamagata), have circulated globally [1]. Influenza vaccine composition is reconsidered bi‐annually to cover the antigenic drift in the haemagglutinin (HA) protein [1]. Until 2012, influenza vaccinations were based on a trivalent vaccine (TIV) composition, with 2 influenza A subtypes (H1N1 and H3N2) and 1 IBV (B/Yamagata or B/Victoria lineage) [2]. To overcome unpredictable circulation of influenza B lineages and frequent vaccine mismatches, quadrivalent influenza vaccines (QIV) were introduced in February 2012, which included both influenza B lineages [3].

B/Yamagata viruses were responsible for a higher proportion of IBV infections than B/Victoria globally in 2012–2017, and a large outbreak of B/Yamagata occurred in 2017 affecting much of the world [4]. However, B/Yamagata viruses showed weak antigenic selection in recent years, reducing their prevalence over time [5] and requiring no update of the vaccine component since 2015 (Table 1). The number of sequences of the B/Yamagata HA genome segment uploaded in influenza surveillance databases showed a major decrease since 2019, in terms of both number of sequences and number of countries reporting. To date, no B/Yamagata viruses have been isolated or sequenced since March 2020 [1, 3].

**TABLE 1:** Influenza B/Yamagata lineage viruses recommended for vaccine composition in Northern Hemisphere (NH) seasons 2012/2013–2024/2025 and in Southern Hemisphere (SH) seasons 2013–2025.

NH season	B/Yamagata strain	SH season	B/Yamagata strain
2012/2013	B/Wisconsin/1/2010‐like	2013	B/Wisconsin/1/2010‐like
2013/2014	B/Massachusetts/2/2012‐like	2014	B/Massachusetts/2/2012‐like
2014/2015	B/Massachusetts/2/2012‐like	2015	B/Phuket/3073/2013‐like
2015/2016	B/Phuket/3073/2013‐like	2016	B/Phuket/3073/2013‐like
2016/2017	B/Phuket/3073/2013‐like	2017	B/Phuket/3073/2013‐like
2017/2018	B/Phuket/3073/2013‐like	2018	B/Phuket/3073/2013‐like
2018/2019	B/Phuket/3073/2013‐like	2019	B/Phuket/3073/2013‐like
2019/2020	B/Phuket/3073/2013‐like	2020	B/Phuket/3073/2013‐like
2020/2021	B/Phuket/3073/2013‐like	2021	B/Phuket/3073/2013‐like
2021/2022	B/Phuket/3073/2013‐like	2022	B/Phuket/3073/2013‐like
2022/2023	B/Phuket/3073/2013‐like	2023	B/Phuket/3073/2013‐like
2023/2024	B/Phuket/3073/2013‐like	2024	B/Phuket/3073/2013‐like
2024/2025	B/Phuket/3073/2013‐like^a^	2025	NA

^a^ For quadrivalent vaccines.

In Italy, influenza B detections predominated over type A in three seasons (2012/2013, 2015/2016 and 2017/2018). Since 2012/2013, viruses of the Yamagata lineage have prevailed over those of the Victoria lineage. During the 2015/2016 season, viruses of the Victoria lineage prevailed again [6]. However, in the 2016/2017 season, there was a return to Yamagata‐lineage viruses [7], dominating in the following 2017/2018 season [8, 9]. In the 2018/2019 season, both B/Yamagata and B/Victoria lineage viruses circulated at very low levels, while in the 2019/2020 season, influenza A and B circulated together, with B/Victoria lineage viruses far outnumbering those of the B/Yamagata lineage. The 2020/2021 and 2021/2022 seasons were strongly affected by the restrictive measures put in place for the pandemic, with low circulation of influenza viruses compared to the prepandemic seasons [10].

All HA gene sequences from the 77 viruses detected in 2020, including 16 from the World Health Organization (WHO) European Region, belonged to genetic clade Yam‐3 and had three HA1 amino acid substitutions (L172Q, D229N and M251V) compared to B/Phuket/3073/2013‐like viruses recommended for use in QIV [11].

Since the start of the COVID‐19 pandemic, there were important changes in the circulation of influenza viruses and other common respiratory infections around the world [1, 3]. Behavioural changes, such as social distancing, mask wearing and hygiene measures, as well as travel and movement restrictions, are thought to be the major factors driving to the reduction of influenza incidence, leading to the possible disappearance of the influenza B/Yamagata lineage [1, 3].

This study aimed at investigating the prevalence of antibodies against B/Yamagata vaccine strain B/Phuket/3073/2013 in the general population and analysing the HA sequences of influenza surveillance isolates for comparison with the reference vaccine strains.

## 2 Methods

### 2.1 Serological Assay

#### 2.1.1 Virus Antigen

The influenza antigen B/Phuket/3073/2013 (B/Yamagata lineage, NIBSC code 21/136) was obtained from National Institute for Biological Standards and Control (NIBSC), propagated in hens' egg and used as native antigen for haemagglutination inhibition assay (HAI).

#### 2.1.2 Serum Samples

Human serum samples were anonymously collected in Italy as residual samples for unknown diagnostic purposes and stored at the laboratory of Molecular Epidemiology of the University of Siena, Italy in compliance with Italian ethics law. For each sample, information on age and year of collection only were recorded. One‐hundred samples were selected for each season from 2012/2013 to 2021/2022, balanced between two age groups, 18–64 years old (younger adults) and ≥65 years old (elderly adults).

Hyperimmune sera of B/Yamagata strains from 2001/2002 season onward were obtained from NIBSC or Influenza Reagent Resource (IRR): B/Guangdong/120/2000 (NIBSC code 01/450), B/Jiangsu/10/2003 (NIBSC code 04/242), B/Florida/4/2006 (NIBSC code 07/356), B/Wisconsin/1/2010 (IRR code FR‐46), B/Massachusetts/2/2012 (NIBSC code 13/182) and B/Phuket/3073/2013 (NIBSC code 15/150).

#### 2.1.3 Haemagglutination Inhibition Assay

A detailed version of the HAI protocol can be found in the FLUCOP project collaborative publication [12]. Two cut‐off values were defined: A serum sample with an HAI titre ≥ 10 was considered positive, while a serum sample with an HAI titre ≥ 40 was considered as protected [13].

#### 2.1.4 Statistical Analysis

The results from HAI were reported as proportion of positive (HAI titre ≥ 10) and protected samples (HAI titre ≥ 40) along different influenza seasons and by age group (18–64 years old and ≥ 65 years old). Median HAI titres along with their interquartile range (IQR) were calculated. Chi‐square test was used to compare proportions of HAI positive and protected samples, and non‐parametric test was used to compare median HAI titres. Statistical significance was set at *p* < 0.05, two tailed.

### 2.2 Sequence Analysis

A total of 156 HA sequences of B/Yamagata viruses was collected in Italy from 2012/2013 to 2017/2018 season in the context of seasonal influenza surveillance. Sequences were collected from two centres, one in Northern Italy (Milan, Lombardy region) and one in Southern Italy (Bari, Apulia region) and released in the Global Initiative on Sharing All Influenza Data (GISAID) (Table 2). No sequences were available for 2018/2019 season and onward.

**TABLE 2:** Sequences collected in Italy from two surveillance centres from 2012/2013 to 2017/2018.

Season	Milan	Bari	Total
2012/2013	17	2	19
2013/2014	NA	NA	NA
2014/2015	26	3	29
2015/2016	3	1	4
2016/2017	11	NA	11
2017/2018	72	21	93

HA sequences of B/Yamagata vaccine strains from 2012/2013 season onward were obtained from GenBank (B/Wisconsin/1/2010, accession number CY115183 and B/Massachusetts/2/2012, accession number KC891816). B/Phuket/3073/2013 HA sequence was referred to EPI_ISL_1760118 isolate.

Sequence alignment was performed using MUSCLE 5.1 and UPGMA tree was generated.

## 3 Results

### 3.1 Serological Study

Out of 1000 samples collected from 2012/2013 to 2021/2022 season, 61.9% of subjects showed HAI antibodies to B/Phuket/3073/2013 virus. Notably, 21.7% had protective antibody levels (HAI titre ≥ 40).

**FIGURE 1:**
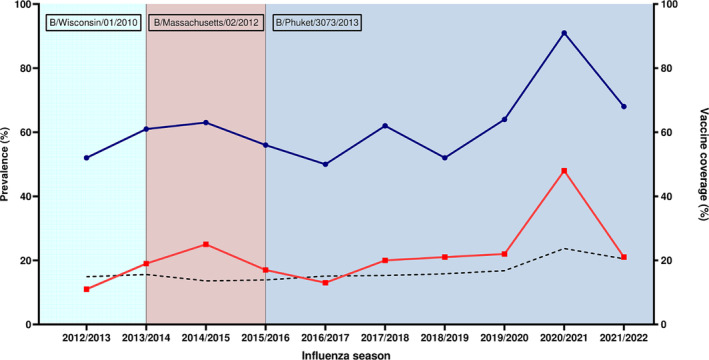
Prevalence of antibodies against influenza B/Phuket/3073/2013 virus from 2012/2013 to 2021/2022 influenza season. Blue line with circles indicates prevalence of HAI titres ≥ 10, while red line with squares indicates prevalence of HAI titres ≥ 40. B/Yamagata vaccine strains by season are indicated in boxes. Dotted line indicates influenza vaccine coverage for general population according to the Italian Ministry of Health [14].

**FIGURE 2:**
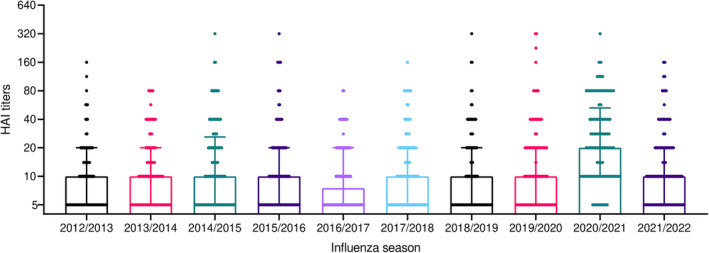
Median HAI titres against influenza B/Phuket/3073/2013 virus from 2012/2013 to 2021/2022 influenza season. Dots represent individual values, and bars represent median with IQR.

The prevalence of the levels of protective HAI antibodies in the seasons from the isolation of the strain and its inclusion in the vaccine was between 11.0% and 25.0%, with no significant changes observed in subsequent years (Figure 1). The only exception is the 2020/2021 season, during which a significant increase to 48.0% of samples showing protective antibody levels was observed (Chi‐square test, *p* < 0.0001), in line with the increase in influenza vaccine coverage during the pandemic. The same was also observed when considering the median HAI titres for each season (Figure 2). The 2020/2021 season was characterized by a significant increase in the median HAI titres (*p* < 0.0001) compared to previous seasons and compared to the subsequent 2021/2022 season as well.

**FIGURE 3:**
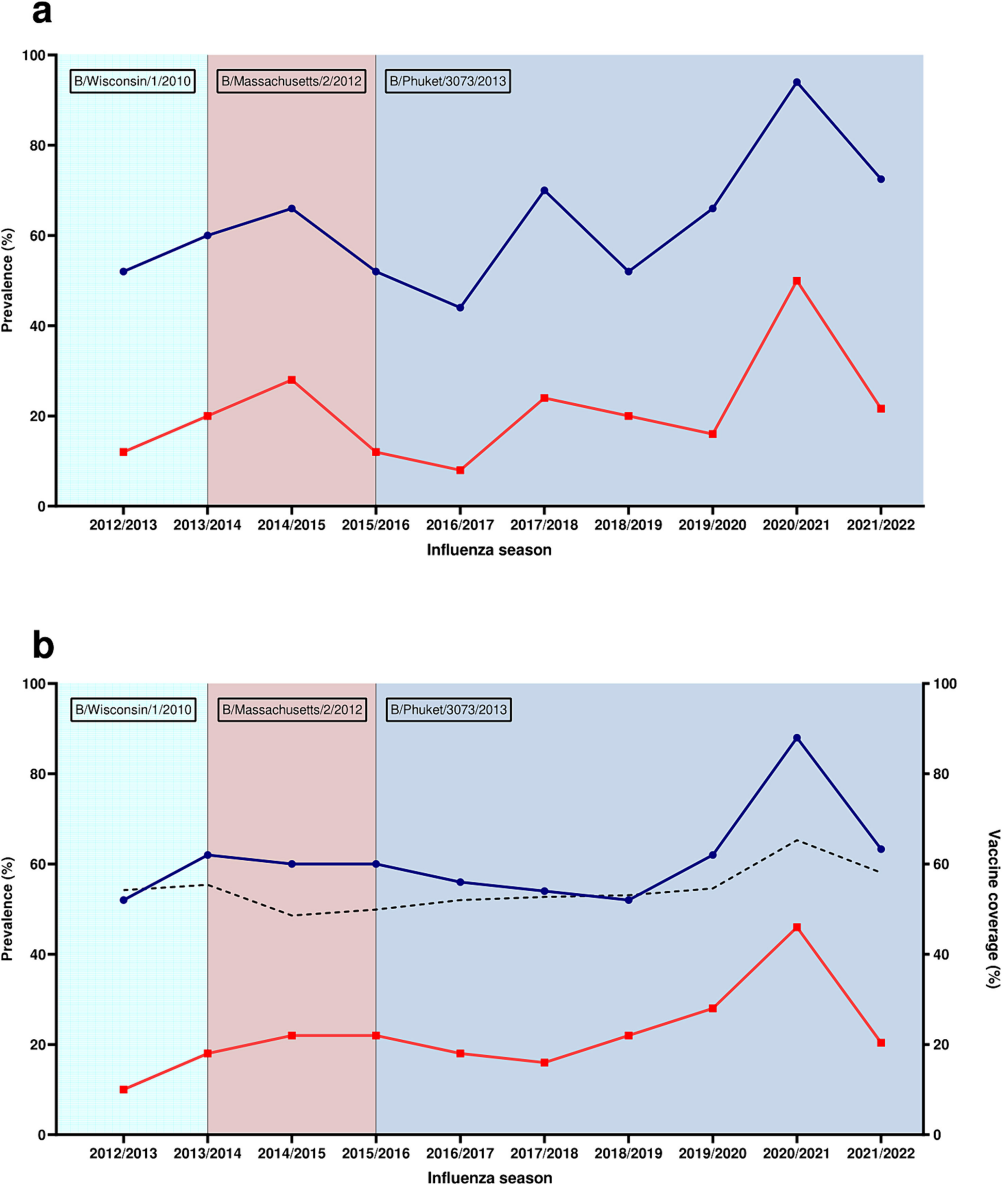
Prevalence of antibodies against influenza B/Phuket/3073/2013 virus from 2012/2013 to 2021/2022 influenza season by age group: 18–64 years old (a) and ≥ 65 years old (b). Blue line with circles indicates prevalence of HAI titres ≥ 10, while red line with squares indicates prevalence of HAI titres ≥ 40. B/Yamagata vaccine strains by season are indicated in boxes. Dotted line indicates influenza vaccine coverage (data available only for elderly) according to the Italian Ministry of Health [14].

**FIGURE 4:**
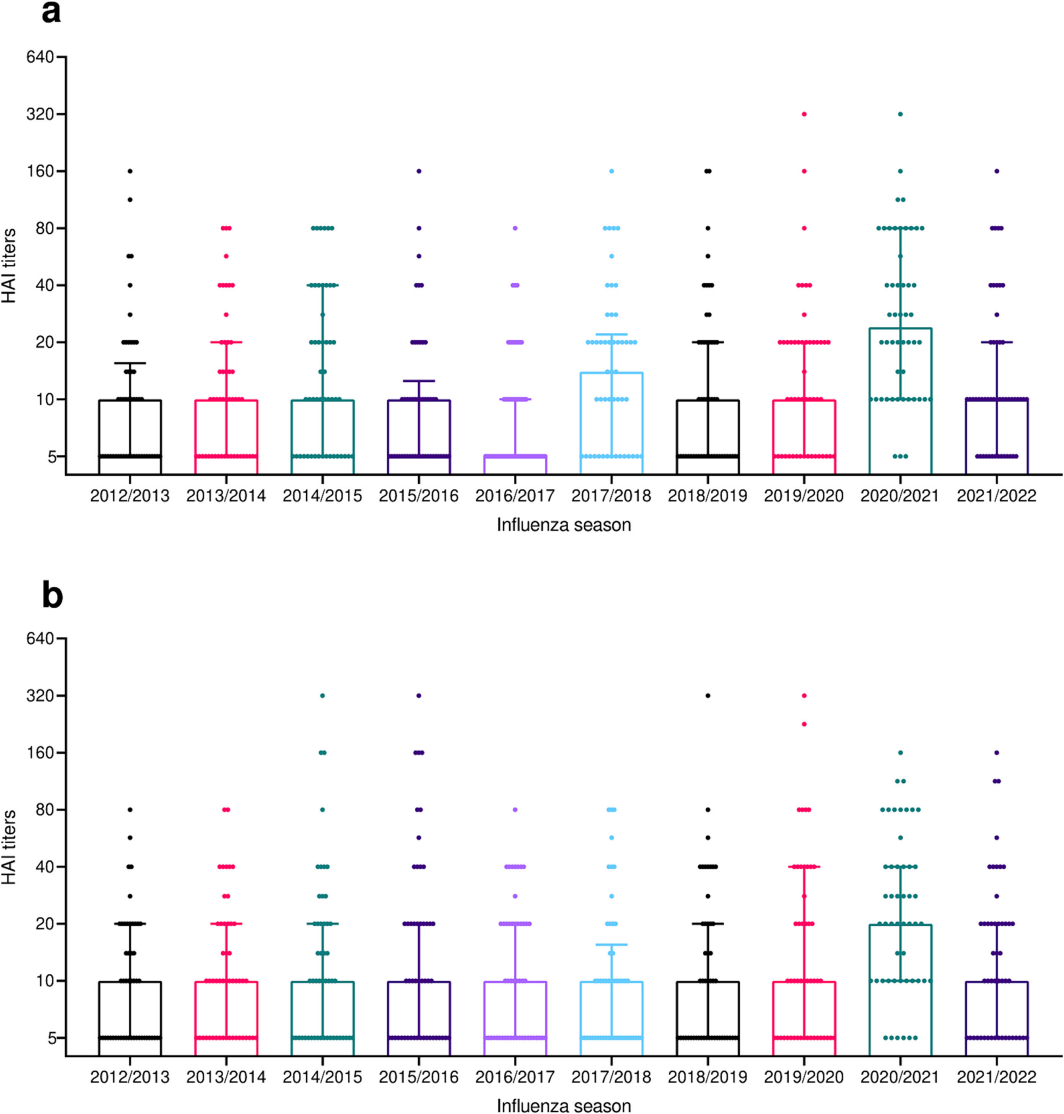
Median HAI titres against influenza B/Phuket/3073/2013 virus from 2012/2013 to 2021/2022 influenza season by age group: 18–64 years old (a) and ≥65 years old (b). Dots represent individual values, and bars represent median with IQR.

Interestingly, results from the two age groups, 18–64 years old (younger adults) and ≥65 years old (elderly adults), showed some differences (Figures 3 and 4). Younger adults showed more variation in prevalence values among the seasons (chi‐square test, *p* < 0.0001), with a significant increase in prevalence not only for the 2020/2021 season but also for the season 2017/2018 (Figure 3a), potentially reflecting the impact of the 2017/2018 influenza B season. The same was also reflected by increased median HAI titres for these two seasons (Figure 4a). In contrast, elderly adults showed a more constant trend, but with a significant increase in 2020/2021 season both in terms of prevalence and HAI titres (Figures 3b and 4b).

B/Yamagata hyperimmune sera showed increasing HAI titres with the chronological succession of seasons, from B/Guangdong/120/2000 (HAI titre of 80) to B/Massachusetts/2/2012 (HAI titre of 640) (Table 3), denoting a certain grade of cross‐reaction respective cross‐protection among B/Yamagata strains.

**TABLE 3:** B/Yamagata hyperimmune sera HAI titres against influenza B/Phuket/3073/2013 strain.

Hyperimmune sera	Clade	HA1 protein homology vs. B/Phuket/3073/2013	HA full protein homology vs. B/Phuket/3073/2013	HAI titre vs B/Phuket/3073/2013
B/Phuket/3073/2013	3	NA	NA	1280
B/Massachusetts/2/2012	2	97.2	98.3	640
B/Wisconsin/1/2010	3	98.9	99.3	640
B/Florida/4/2006	1	97.8	98.5	320
B/Jiangsu/10/2003	1	95.8	97.4	40
B/Guangdong/120/2000	1	92.7	92.7	80

Abbreviation: NA, not applicable.

### 3.2 Sequence Analysis

Alignments of HA nucleotide sequences were performed. Considering the B/Yamagata‐like strains that circulated during the 2012/2013 season, a high proportion (89.5%) clustered to the Yam‐2 clade, related to the B/Massachusetts/2/2012 strain. The only exception is represented by two isolates that clustered in the Yam‐3 clade, closely related to the B/Wisconsin/1/2010 strain. From 2014/2015 season onward, B/Yamagata isolates clustered in the Yam‐3 clade, closely related to the B/Phuket/3073/2013 strain.

**FIGURE 5:**
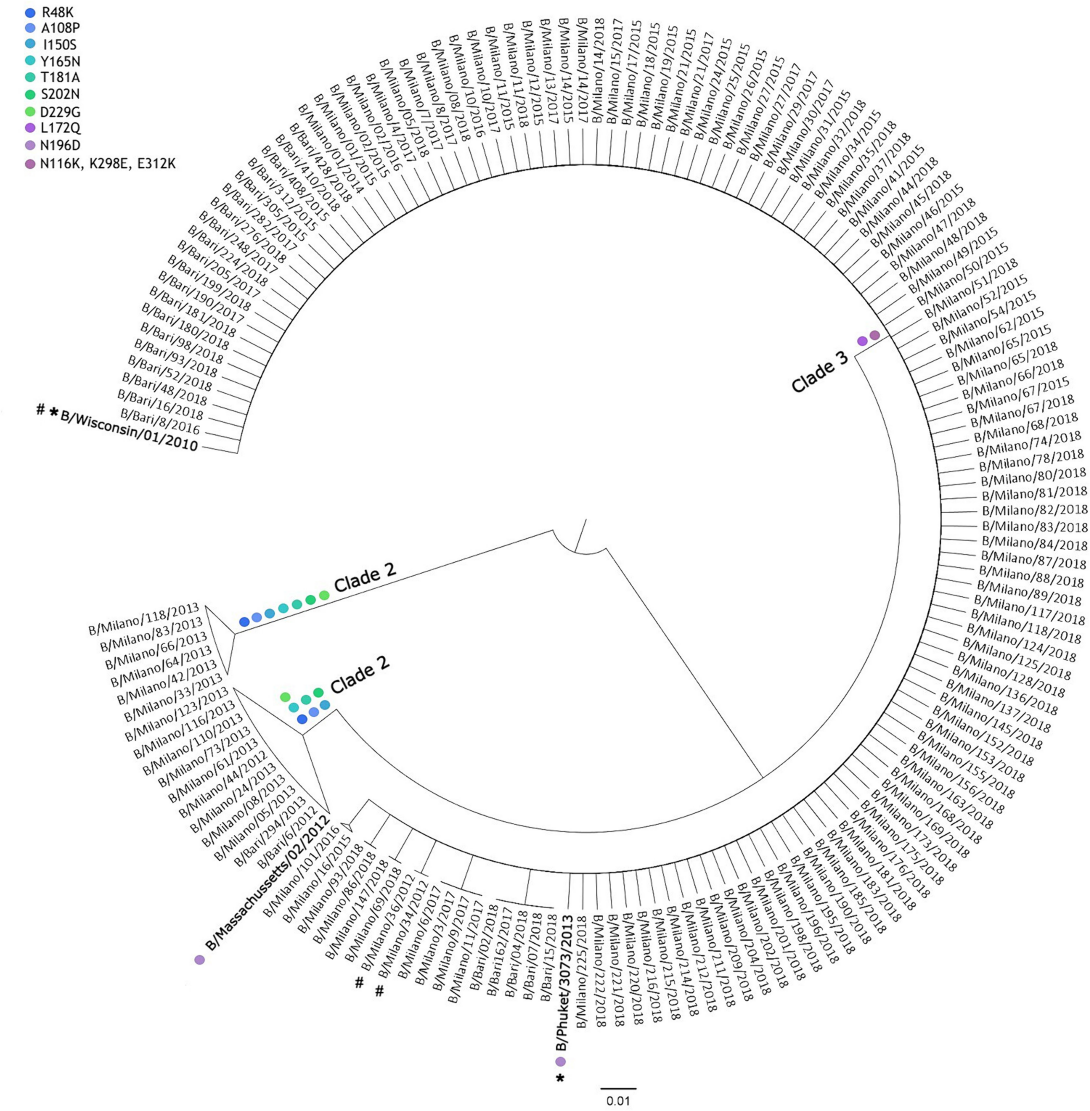
Phylogenetic tree of the HA protein sequences from influenza B/Yamagata lineage viruses detected in Italy from 2012/2013 to 2017/2018. The analysis includes 156 sequences of isolates and the three recommended vaccine strain (B/Wisconsin/1/2010, B/Massachusetts/2/2012, B/Phuket/3073/2013, marked in bold). Clades were marked and mainly characterizing amino acid substitutions in comparison with B/Wisconsin/1/2010 vaccine strain were reported (coloured dots, see legend in the figure). * not showing the L172Q substitution; # not showing N116K, K298E, E312K substitutions.

These results reflect the performed alignments on HA protein sequences (Figure 5). With the exception of two of them, all isolates from season 2012/2013 showed high homologies to the HA of the B/Massachusetts/2/2012 strain, with the majority of amino acid changes in the four major epitope domains of HA1, comprising the 120‐loop (position 116–137), the 150‐loop (position 141–150), the 160‐loop (position 162–167) and the 190‐helix (position 194–202) and their surrounding regions. In particular, seven amino acids substitutions were observed (R48K, A108P, I150S, Y165N, T181A, S202N, D229G) compared to the vaccine strain recommended for the 2012/2013 (B/Wisconsin/1/2010). However, the two isolates of the 2012/2013 showed a high homology to B/Wisconsin/1/2010 strain.

The following seasons were characterized by B/Phuket/3073/2013‐like viruses. When comparing with the vaccine strain B/Phuket/3073/2013, a series of amino acid changes were observed in the HA protein. In particular, all isolates showed the L172Q substitution and, massively in 2017/2018 season, M251V, characterizing B/Phuket/3073/2013‐like isolates with respect to the B/Phuket/3073/2013 vaccine strain. A gradual drift was observed, allowing to identify subgroups into the lineage, reporting other changes such as D229N, K211R, V176I and S207P.

## 4 Conclusions

With respect to the potential extinction of the B/Yamagata lineage, this study aimed to investigate the prevalence of antibodies against the B/Yamagata vaccine strain B/Phuket/3073/2013 in the general Italian population.

The results from this serological study show a consistent prevalence of antibodies against B/Yamagata viruses circulating during almost a decade. The only exception is the 2020/2021 season with significant increase in subjects with sero‐protective antibody levels which is in line with the increase of influenza vaccine coverage during the pandemic [14].

During a decade, from 2012/2013 to 2021/2022, about 21.7% of subjects had protective antibody levels against B/Phuket/3073/2013, despite of its introduction as vaccine strain for the last seven influenza seasons. Instead of reaching a high plateau of immunity population, antibodies seem to wane, in line with antibody titres decline observed over a season [15, 16]. This population level decline in antibody prevalence and antibody titres may be a sign for missing constant boostering by missing contact with B/Yamagata strains.

Hyperimmune sera for B/Yamagata viruses of the past 20 years show increasing levels of cross‐reactivity against the B/Yamagata‐lineage vaccine strains B/Phuket/3073/2013 over time, consistent with a more gradual antigenic variation [17].

Clinical trials of vaccination against IBV in children and experimental infection of mice [18, 19, 20] have shown that the immune response is more robust towards B/Yamagata antigens than towards B/Victoria ones. This indicates that B/Yamagata viruses evolve less through antigenic drift and therefore less able to escape the immune response [21]. Furthermore, B/Victoria antigen has shown to be an effective immunogen for a cross‐lineage boost [18]. Consequently, it is possible that, in the presence of sufficient immune coverage such as in the case of QIV use, the prevalence of B/Yamagata viruses might decline sharply over time [21].

Nevertheless, in the absence of a known threshold necessary for the extinction of a lineage, it is difficult to pose whether the overall immunity in the population was sufficient to cause the extinction of the B/Yamagata lineage.

The slow evolutionary dynamics exhibited by B/Yamagata viruses also emerges from sequence analysis of B/Yamagata isolates from surveillance activities. A high proportion (89.5%) of B/Yamagata strains circulating in Italy during the 2012/2013 season were related to Yam‐2 clade (B/Massachusetts/2/2012‐like strains) and antigenically distinct from the B/Wisconsin/1/2010 (Yam‐3 clade) vaccine strain recommended for the season.

For this reason, B/Massachusetts/2/2012 was included in the vaccine formulation for the subsequent 2013/2014 and 2014/2015 seasons, although a shift to the Yam‐3 clade, closely related to B/Phuket/3073/2013, was observed. This latter strain differed from the B/Massachusetts/2/2012 vaccine strain by 10 amino acid substitutions in the HA1 and showed N116K, K298E and E312K changes compared to B/Wisconsin/1/2010.

From 2015/2016 season onward, only B/Yamagata viruses belonging to the Yam‐3 clade circulated, showing limited amino acid variation. All the viruses clustered together with the 2015/2016 B/Phuket/3073/2013 vaccine strain, with the additional L172Q, D196N and M251V amino acid changes.

In recent seasons, B/Yamagata isolates have thus shown only limited amino acid variation in the HA, limiting the role played by immune selection on this protein in the recent evolution of this lineage. The B/Yamagata viruses circulating in 2017–2019 formed a single monophyletic lineage (clade 3A), and only recently, a subgroup of viruses from clade 3A acquired additional HA mutations (i.e., K211R and D229N). However, none of these amino acid substitutions have clear antigenic effects using post‐infection ferret antisera [11]. The antigenic characterization of recent viruses belonging to the Yam‐3 clade shows good cross‐reactivity with the vaccine strain of the B/Yamagata lineage (B/Phuket/3073/2013‐like), which has remained unchanged since 2015 [22].

As of 23 January 2024, no B/Yamagata lineage viruses have been detected after March 2020 or sequences released in GISAID [23, 24].

As influenza B activity is highly variable worldwide, the disappearance of one or even both lineages in specific seasons could be related to the genetic variability of the IBV [25, 26]. However, the prolonged use of a well‐matched influenza vaccine and the low antigenic diversity of B/Yamagata viruses in recent years may have favoured a strong reduction in B/Yamagata circulation. This, combined with the restrictive conditions of the COVID‐19 pandemic, especially in the seasons 2020/2021 and 2021/2022, may have potentially led to the extinction of this lineage [1].

In China, IBV dominated after the COVID‐19 pandemic, with 99.7% of B/Victoria viruses in the 2020/2021 season. The possible extinction of B/Yamagata viruses, as well as the decrease in influenza A cases, is assumed to be related to differences in susceptibility to infection by different groups of population. Adults and the elderly were more affected by the restrictive measures during the pandemic, interrupting the transmission of B/Yamagata viruses [27].

The current disappearance of B/Yamagata will have important implications for the use of QIVs, which are common in many regions and countries globally, for example, Australia, Canada, Europe, Japan and the United States [28, 29, 30]. According to the WHO influenza vaccine composition advisory committee, B/Yamagata lineage should be excluded from influenza vaccines, urging national authorities to make decisions regarding the return to TIV [31, 32]. Furthermore, with the extinction of the B/Yamagata lineage, there is the possibility for its subsequent re‐introduction, which (1) poses a risk to the population in the years to come, since if the lineage returns, it is unlikely to be antigenically similar to the virus now included in the QIV [33], and (2) opens up the question of the level of safety for its handling, due to the potential risk of an extinct influenza virus escaping [3, 4].

As IBV are mostly restricted to humans, high vaccination coverage inducing broad immune protection could support their eradication [1]. The significant reduction in global influenza virus circulation due to the COVID‐19 pandemic, combined with the use of QIV and a low antigenic diversity of B/Yamagata viruses, may have resulted in the extinction of the B/Yamagata lineage.

## Author Contributions

Conceptualization: S.M., N.T., C.M.T. Formal analysis: S.M., M.B. Funding acquisition: E.M. Investigation: S.M., G.M. Project administration: S.M. Resources: I.M., M.C., E.P., E.M., C.M.T. Data curation: S.M. Supervision: C.M.T. Visualization: S.M. Writing – original draft preparation: S.M. Writing – review and editing: M.B., G.M., I.M., M.C., E.P., A.M., O.K., E.M., N.T., C.M.T.

## Ethics Statement

All experiments were performed in compliance with relevant laws and institutional guidelines and in accordance with the ethical standards of the Declaration of Helsinki.

## Conflicts of Interest

A.M. is employed by VisMederi srl. E.M. is founder and Chief Scientific Officer of VisMederi srl and VisMederi Research srl. C.M.T. is an external consultant of VisMederi srl and VisMederi Research srl. O.K. is an external consultant of VisMederi srl.

## Consent

Written informed consent was obtained from all subjects.

## Data Availability

The original contributions presented in the study are included in the article, and further inquiries can be directed to the corresponding author.
